# Sintering Kinetics of Austenitic Stainless Steel AISI 316L Modified with Nanographite Particles with Highly Developed BET Specific Surface Area

**DOI:** 10.3390/ma13204569

**Published:** 2020-10-14

**Authors:** Barbara Kozub, Jan Kazior, Aneta Szewczyk-Nykiel

**Affiliations:** Faculty of Materials Engineering and Physics, Institute of Materials Engineering, Cracow University of Technology, Al. Jana Pawła II 37, 31-864 Cracow, Poland; jkazior@pk.edu.pl (J.K.); anykiel@mech.pk.edu.pl (A.S.-N.)

**Keywords:** sintering kinetics, nanographite particle, BET specific surface area, sintered stainless steel, AISI 316L

## Abstract

The subject of this work was the study of processes occurring during sintering of water atomized AISI 316L austenitic stainless steel powder modified by the addition of graphite nanoparticles. The main purpose of the work was to determine the effect of modification of the AISI 316L stainless steel austenitic powder by the addition of graphite nanopowder on the sintering kinetics and oxide reduction mechanism. The phenomena occurring during the sintering process and oxide reduction mechanisms were subjected to detailed characterizations. Mixtures with two types of nanopowder with a high BET (measurement technique of the specific surface area of materials based on Brunauer–Emmett–Teller theory) specific surface area of 350 and 400 m^2^/g and for comparison with graphite micropowder with a poorly developed BET specific surface area of 15 m^2^/g were tested. The conducted thermal analysis showed that the samples made of austenitic stainless steel doped with 0.2% and 0.3% by weight graphite nanopowder with a BET specific surface area of 400 m^2^/g, sintered best the oxide reduction reactions, with a more intensive participation of carbon, for these samples.

## 1. Introduction

Powder metallurgy is a technology involving the production of metallic powders and products from these powders and their mixtures with non-metals or with alloy or partially alloyed powders, using forming and sintering processes. Techniques for producing sintered machine parts include two basic operations, i.e., compacting of powder, most often in specially profiled dies to form shaped bodies with specific geometry, and then sintering of these shaped compacts. A characteristic feature of powder metallurgy technology is the fact that the entire product does not need to be passed through the liquid state during the production process, thus compacts retain their shape during the entire technological cycle, with possible slight shrinkage or swelling of the moldings [1,2,3,4,5,6,7,8].

In the case of stainless steel powders, the necessary condition for starting the sintering process is the reduction of the oxide coating formed on the surface of the powder particles. AISI 316L stainless steel owes its very good corrosion resistance to high chromium and molybdenum content. Chromium oxides—Cr_2_O_3_ forming a coherent layer protecting the surface of powder particles against corrosion are classified as very stable and difficult to reduce oxides. In turn, molybdenum oxides increase the corrosion resistance of steel under operating conditions at elevated temperatures [9,10,11,12,13,14,15,16]. Due to the high chromium content (Cr–17%) and very high stability of chromium oxides, it is their elimination that is a prerequisite for starting the sintering process. Silicon oxide—SiO_2_ is another very stable oxide that must be removed during sintering. These oxides in austenitic stainless steels are a residue from the atomization process. Silicon, due to its high affinity for oxygen, is added to steel to prevent chromium oxidation [1]. Elimination of the tight oxide layer formed on the surface of the AISI 316L stainless steel powder particles, depending on the sintering atmosphere used, can occur in two ways by hydrogen reduction or by thermal decomposition in a vacuum [1,10,11,12,13,14,15,16,17,18]. Based on the analysis of the plot of hydrogen partial pressure versus partial pressure of water vapor as a function of temperature for various metal oxides available in the literature [19], it can be determined whether individual oxides will be reduced, for the assumed parameters of the sintering process (i.e., temperature, atmosphere). On analyzing these graphs, it can be stated that during the sintering process in a vacuum atmosphere (for partial oxygen potential of P_O2_ = 10^−2^ Pa), the most stable oxides, i.e., Cr_2_O_3_ and SiO_2_, will not be reduced. The situation is different in the case of sintering in an atmosphere of pure hydrogen, which has a high reducing potential. At a temperature of 1280 °C and a ratio of hydrogen partial pressure to water partial pressure equal to P_H2/H2O_ = 9.56∙10^4^ Pa, chromium oxides can be reduced, but the reduction of silicon oxides present on the surface of AISI 316L stainless steel austenitic powder particles may be difficult.

In their studies, German and Madan [20,21] presented a model of the activated sintering of iron, based on which, under certain conditions, an appropriate choice of the sintering activator can be made. These studies show that elements with a low atomic number (e.g., boron, carbon, nitrogen) and flame-retardant elements (such as molybdenum, tungsten, and tantalum) show a high ability to activate the sintering process. The model presented by German and Madan is based on three basic criteria for activator selection: segregation criterion—the activating additive should segregate across grain boundaries, solubility criterion—the activator must be characterized by low solubility in the matrix, a requirement of high solubility of the matrix in the activator with diffusion criterion—easy path of diffusion of matrix atoms through the segregated phase at the grain boundary [20,21].

Many authors, including Kazior et al. [1], Molinari et al. [22], Terrisse et al. [10], Tunberg et al. [23], Skałoń et al. [24], Larsen et al. [12], Sulikowska et al. [25] and Danninger et al. [16], conducted research that showed that in the case of sintering austenitic stainless steels, introduction of the appropriate alloying additives to the chemical composition can activate and increase the kinetics of the sintering process itself. In their work, the authors mainly focused on the use of carbon and boron as alloying additions. Kazior in the work “Boron in sintered austenitic stainless steels” broadly describes the effect of the addition of boron on the structure and properties of sintered austenitic stainless steels [26], which allows on appearance of the liquid phase activation of the sintering process.

In their works, Molinari et al. [22,27], Terrise et al. [10], Tunberg et al. [23], Larsen et al. [12], Bergman et al. [15], Danninger et al. [17] presented an alternative for sintering in a reducing atmosphere containing, for example hydrogen, a method consisting of introducing graphite powder into metal powder before pressing and subsequent sintering of the compacts in a vacuum. Carbon reduces the oxide layer to form carbon monoxide—CO, which then allows the reduction of oxides to form carbon dioxide—CO_2_ [10,11,12,13,14,15,16,23,28].

When using this method, it should be remembered that the amount of graphite added must be relatively small, due to the risk of formation of Cr_23_C_6_ chromium carbide precipitations at the grain boundaries during cooling, which result in a local, significant reduction of corrosion resistance [23]. Therefore, the amount of carbon in sinters should not exceed 0.03% by weight—this value for low- carbon austenitic stainless steels is considered to be the limit.

Larsen and Thorsen [12] showed in their work that graphite added to 316L stainless steel in the amount of 0.1% and 0.3% by weight is a good activator of reduction processes during sintering in a vacuum. However, the temperature profiles adopted by the authors assumed a very long sintering process, which is not economically advantageous. The sintering process in the article presented by Larsen and Thorsen consisted of a firing stage at 500 °C for 60 min and a sintering at 1150 °C for 240 min or at 1250 °C for 120 min. The test results obtained by them are similar-in terms of intensification of the reduction reaction as well as carbon and oxygen content in sinters to the results obtained for the tested sintered steel in a reducing atmosphere of hydrogen.

In their research, Terrise et al. [10] focused on determining the impact of vacuum height and sintering temperature on the reduction of oxides occurring in austenitic stainless steel. The obtained results showed that in the case of sintering in a vacuum of 10^−3^ mbar, the oxide layer was completely reduced at 1000 °C. However, for a vacuum of 10^−5^ mbar, the reduction of the oxide coating already occurs at a temperature of 800 °C. Unfortunately, the authors did not examine the mechanical properties and the corrosion resistance of sinters in their work—they refer only to reduction processes and measurements of oxide layer thickness in sinters. Their test results showed the presence of an oxide layer in all tested sinters. The authors explained the presence of the oxide layer as being formed by the oxidation of sinters in the air after the sintering process. However, it was emphasized that the thickness of this layer was smaller the higher the vacuum applied during sintering.

Tunberg, Nyborg, and Liu were the next researchers who took up the subject of sintering of austenitic 304L stainless steel modified with graphite in a vacuum [23]. In their work, the authors showed that the addition of graphite improved the sintering process by intensifying the oxide reduction reaction and improving the strength properties of the tested steels. The amount of 0.19% by weight of graphite added into 304L steel was determined as optimal. However, for the other tested compositions, the obtained results were not satisfactory.

In turn, Toennes and German [11] examined the effect of the addition of graphite and boron on the density and microstructure of sintered SS 422 stainless steel. Their test results showed that the addition of boron—in the case of the tested steel and the adopted sintering parameters (sintering temperature 1320 °C, sintering time 60 min)—had a much better effect on the sintering process, compared to graphite, resulting in a better densification of sinters. In addition, the study states that in order to achieve better results for graphite-modified SS 422 steel, the temperature of isothermal sintering should be further increased.

In their work, Padmavathi et al. [29] presented the effect of sintering temperature, sintering type, and amount of graphite addition on the compaction and corrosion resistance of austenitic 316L stainless steel and 434L ferritic stainless steel. Sintering was carried out by two methods: conventional (convection heating) and microwave method. The amount of graphite introduced into the tested stainless steels was 0.5%, 1.0%, and 1.5% by weight. The authors sintered for two temperatures of 1200 °C and 1300 °C in a hydrogen atmosphere. The addition of graphite for both steels improved the density and corrosion resistance of the sinters. In addition, the isothermal sintering temperature used had a significant impact on the density and corrosion resistance of the sinters tested—they reached higher values when the sintering temperatures were adopted. In the case of ferritic steel, the type of sintering did not have a significant impact on the density and corrosion resistance of sinters. It was different in the case of austenitic stainless steel, for which conventional sintering did not bring satisfactory results—the density and corrosion resistance of the sinters were the lowest.

Also Tizinai et al. [30] examined the influence of vacuum sintering on mechanical properties of copper alloyed austenitic stainless steel. 

In their works, Bergman et al. [15] and Danninger et al. [16] confirmed that carbothermal reduction—represented by large CO peaks registered during quadrupole mass spectrometry (QMS)—is the main mechanism for reducing oxides in the sintering process of high chromium steels doped with graphite.

The main purpose of the work was to determine the effect of modification of AISI 316L stainless steel austenitic powder by the addition of nanographite powder on the sintering kinetics and oxide reduction mechanism. Based on the review of the literature, it can be concluded that in the subject of sintering in the atmosphere of a technical vacuum, fittings made of graphite-doped AISI 316L stainless steel, there is a research gap as no publicly available test results use graphite in the form of nanopowder with a high degree of BET (measurement technique of the specific surface area of materials based on Brunauer–Emmett–Teller theory [31]) specific surface area development as a modification agent for sintered AISI 316L stainless. Moreover, the soundness of conducting such tests is confirmed by the above mentioned literature reports which indicate that the addition of micrographite powder to stainless steels does not always—depending on the choice of its quantity and sintering parameters—allow satisfactory results to be achieved in the reduction of oxides during the sintering process. However, in the case of nanographite powders, due to their highly developed BET specific surface area, it is suspected that the reduction reaction will intensify during sintering, which will allow for faster exposure of pure metallic surfaces, resulting in a reduction of porosity. Additionally, the possibility of sintering fittings made of AISI 316L stainless steel doped with graphite in an atmosphere of a technical vacuum will eliminate the need to use a reducing atmosphere, containing e.g., hydrogen—considered dangerous and not always possible to use in industrial conditions.

## 2. Materials and Methods

### 2.1. Materials

**Figure 1:**
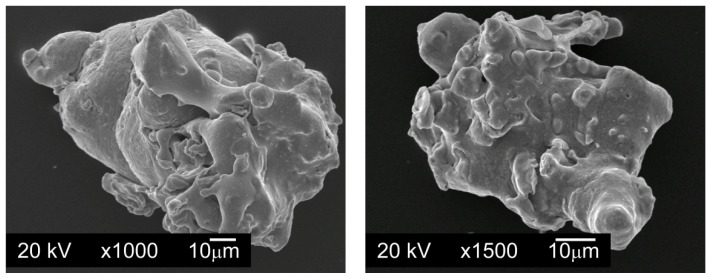
The powder particles of water atomized AISI 316L austenitic stainless steel powder, manufactured by Höganäs AB, SEM.

To carry out the tests, powder mixtures were made in which the base material was water atomized AISI 316L austenitic stainless steel powder, manufactured by Höganäs AB, Sweden (Figure 1). The chemical composition of AISI 316L powder is shown in Table 1. The tested powder has a flowability of 29.1 s/50 g and a bulk density of 2.90 g/cm^3^. 

**Figure 2:**
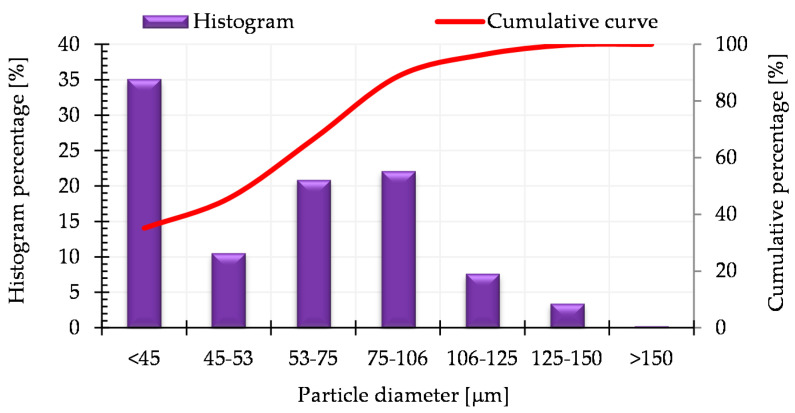
Histogram of particle size distribution and cumulative particle size distribution curve for water atomized AISI 316L austenitic stainless steel powder, manufactured by Höganäs.

Figure 2 presents the histogram of the particle size distribution and the cumulative particle size distribution curve for the AISI 316L austenitic stainless steel powder (the data was provided by the manufacturer in the certificate for the powder analysis).

**Figure 3:**
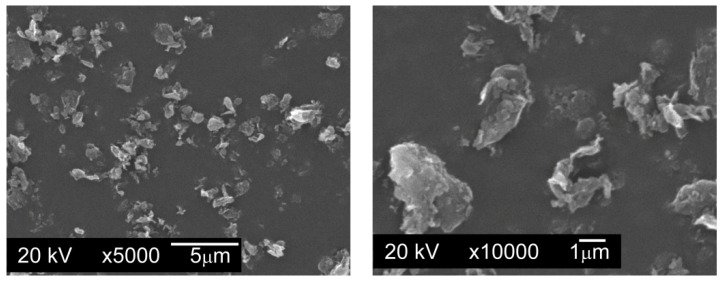
Particles of graphite nanopowder with a BET (measurement technique of the specific surface area of materials based on Brunauer–Emmett–Teller theory [31]) specific surface area of 350 m^2^/g and range of particle size from 0.20 µm to 20 µm, SEM.

**Figure 4:**
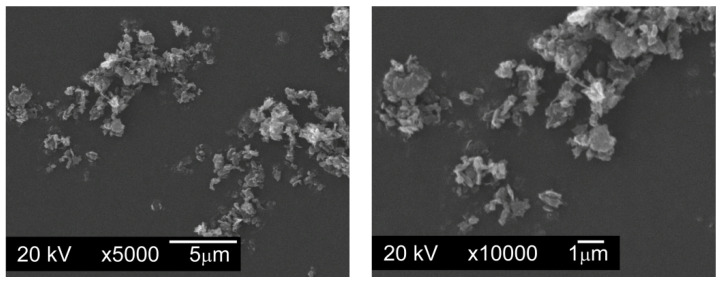
Particles of graphite nanopowder with a BET specific surface area of 400 m^2^/g and range of particle size from 0.25 µm to 5.01 µm, SEM.

**Figure 5:**
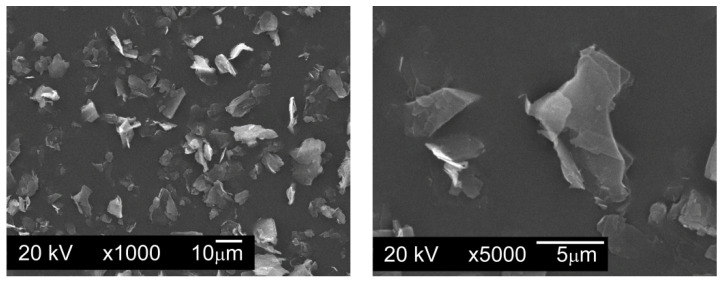
Particles of graphite micropowder with a BET specific surface area of 15 m^2^/g and range of particle size from 6.8 µm to 27.2 µm, SEM.

Modifying additives introduced in quantities of 0.1%, 0.2%, and 0.3% by weight were the following graphite powders:

- Graphite GS-TC307—graphite nanopowder from Graphitestore.com (Northbrook, IL, USA) with a nominal BET specific surface area of 350 m^2^/g and range of particle size from 0.20 µm to 20 µm (Figure 3),
- Graphite GS-2299—graphite nanopowder from Graphitestore.com (Northbrook, IL, USA) with a nominal BET specific surface area of 400 m^2^/g and range of particle size from 0.25 µm to 5.01 µm (Figure 4),
- TIMREX F10 PM Special Graphite—flake graphite micropowder from TIMCAL SA (Bodio, Switzerland), with a nominal BET specific surface area of 15 m^2^/g and range of particle size from 6.8 µm to 27.2 µm (Figure 5).

**Figure 6:**
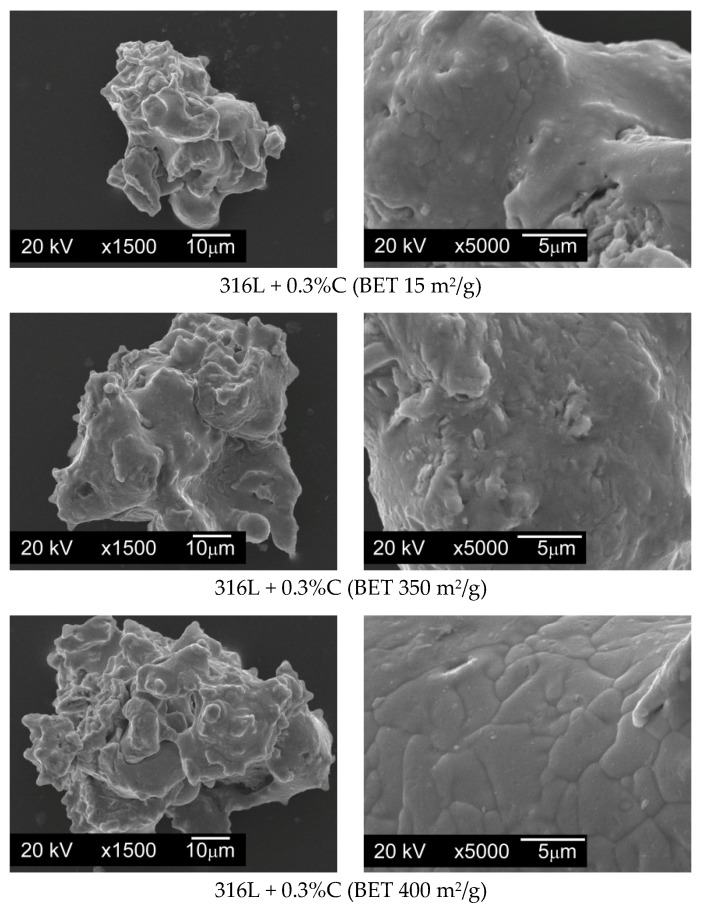
Particles of AISI 316L austenitic stainless steel powder after mixing with graphite micro- and nanopowder addition.

When preparing mixtures, graphite powders were added to the base powder, and then mixed for 12 h using a Turbula T2F mixer (Glen Mills Inc., Clifton, NJ, USA) for loose materials. In order to check whether the graphite particles evenly coated the surfaces of the AISI 316L steel powder particles without forming agglomerates, SEM images were taken for all powder mixtures made, using a JSM5510LV scanning electron microscope with EDS attachment (JEOL Ltd., Tokyo, Japan). Figure 6 presents photomicrographs of powder particles for exemplary mixtures.

On the basis of the taken photos of all the prepared mixtures it can be stated that the whole of the graphite was evenly spread on the surface of the austenitic stainless steel particles and no carbon agglomerates were observed.

Table 2 presents the compositions of the prepared mixtures and their designations.

### 2.2. Testing Methods

The density of compacts and sinters was determined by the geometric method, based on measuring the mass and dimensions of the samples (ten repetitions of measurements were made, on the basis of which the standard deviation was determined). In order to determine the apparent density, total porosity, as well as open and closed porosity of sinters, measurements were carried out using the Archimedes method (PN-EN ISO 2738: 2001 [33]).

In order to examine the mechanisms that occur during the sintering process, dilatometric tests (DIL) were carried out on a 402 PC horizontal dilatometer (NETZSCH-Gerätebau GmbH, Selb, Germany). For the purposes of the tests, cuboidal 4 × 4 × 15 mm^3^ samples were made, pressed on the WWe-100 V.1 hydraulic press (Otto HS, Reda, Poland) with a pressure of 600 MPa. The sintering process was carried out in a technical vacuum (10^−2^ Pa). Isothermal sintering was carried out for temperatures of 1200 °C, 1240 °C and 1280 °C for 30 min. The heating and cooling speed of the samples was 10 °C/min. 

Complementing dilatometric tests, allowing conclusions to be drawn regarding mechanisms occurring during sintering of the tested materials consisted of thermogravimetry (TG) combined in one process with differential scanning calorimetry (DSC) coupled with a quadrupole mass spectrometer (QMS), carried out on an STA 409CD device (NETZSCH-Gerätebau GmbH, Selb, Germany). The tests were carried out for three temperatures: 1200 °C, 1240 °C, and 1280 °C, in an argon atmosphere with a purity of 99.999%, with a heating and cooling rate of 10 °C/min.

Due to the variety of forms of occurrence in the sample, the analysis of the elements in metals, ceramics and other materials, is one of the most difficult analytical tasks. In order to determine the oxygen and carbon content in the sinters tested, an analysis was performed on an RO-416 DRRO-416DR apparatus and the CS-125 apparatus (LECO Corporation, St. Joseph, MI, USA) (three repetitions of measurements were made, on the basis of which the standard deviation was determined).

The measurement of oxygen content in sinters was carried out in two stages: gas evolution from the sample and an analysis of the separated gases. The test begins after placing the graphite crucible between two furnace electrodes. The high current generated, flowing through the crucible, generates a large amount of heat, which causes degassing of the crucible. The sample is then placed in the crucible, which is melted in a protective atmosphere of helium 5.0 (99.999%). Simultaneously, helium carries gases released from the sample. During the melting process, oxides react with carbon from the crucible, turning into carbon monoxide CO, which through the catalyst (CuO based on rare earth elements), oxidizes to CO_2_. In the second stage of the analysis, the extracted gas quickly travels from the liquid metal to the measuring part of the apparatus, where the oxygen content is determined. In the RO-416 DRRO-416DR, oxygen is determined by infrared adsorption (IR), where heteroatom gas molecules (in this case CO_2_) exhibit a characteristic absorption spectrum in the infrared. After leaving the IR cell, CO_2_ is removed by askarite and anhydron.

In order to determine the part of the carbon remaining in the sinters after the sintering process in a vacuum atmosphere, measurements of the carbon content in the sinters were carried out. Measurements were made on a CS-125 apparatus enabling measurement of carbon content in non- ferrous alloys, steels, and solid non-metallic materials. The measurement consisted of burning in a high-frequency induction furnace, in a stream of pure oxygen, the sample placed in a ceramic crucible, followed by a measurement of the infrared absorption coefficient by CO_2_.

## 3. Results

### 3.1. Green and Sintered Density

**Figure 7:**
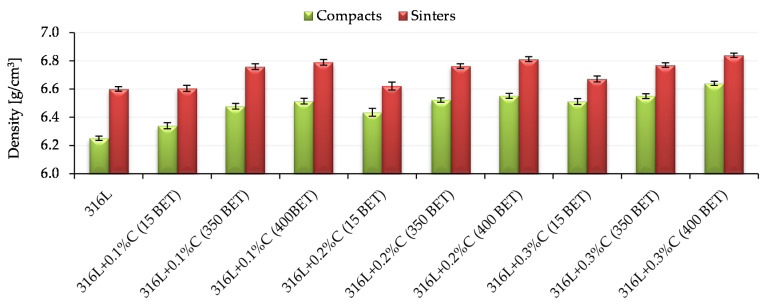
Density of compacts and sinters depending on the percentage composition of mixtures, pressing pressure 600 MPa, isothermal sintering temperature 1280 °C.

Figure 7 presents sample results of density measurements of compacts and sinters made of AISI 316L austenitic stainless steel powder and mixtures of this powder with graphite in the form of micropowder, nanopowders with a BET specific surface area of 350 m^2^/g and 400 m^2^/g. All tested samples were pressed on one side with a pressure of 600 MPa. The results shown in Figure 7 were obtained for samples sintered at the temperature 1280 °C. Standard deviations are marked on the bars presented in Figure 7.

Based on the analysis of the all obtained test results, it can be stated that both the type of additive used and its quantity had a significant impact on the obtained densities of compacts and sinters. In the case of samples made of graphite-doped stainless steel, the following tendency can be observed—the greater the proportion of the addition of graphite and the greater the degree of development of a BET specific surface area of its particles, the higher the values of the densities obtained, both in the case of compacts and sinters. For both compacts and sinters, the highest densities were obtained for samples made of a mix of austenitic stainless steel powder with the addition of 0.3% by weight of graphite nanopowder with a BET specific surface area of 400 m^2^/g. It is worth noting that an equally high density value after sintering was obtained for a sample made of AISI 316L powder doped with graphite nanopowder with a specific surface area of 400 m^2^/g, added in an amount of 0.2% by weight. In the case of sintered samples at lower temperatures, i.e., 1200 °C and 1240 °C, the obtained densities of compacts and sinters had lower values, however, the obtained tendencies were of the same nature as in the case of sintered samples at 1280 °C.

**Figure 8:**
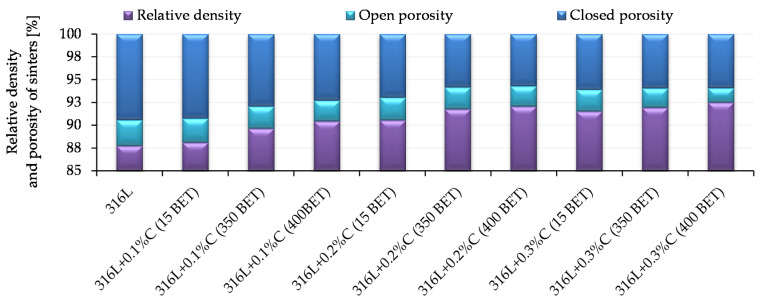
Relative density and porosity of sinters depending on the percentage composition of the mixture, pressing pressure 600 MPa, isothermal sintering temperature 1280 °C.

Archimedes density measurements were carried out for samples pressed at 600 MPa and sintered at 1280 °C, for which the highest density values measured by the geometric method were obtained. The obtained results of measurements of relative density and porosity of sinters, for all tested materials, are presented in Figure 8.

In all analyzed cases, the total porosity in the majority consists of closed porosity, which is important from the point of view of mechanical properties and corrosion resistance of sintered steel. As in the case of the density measured by the geometric method, the value of total and open porosity was influenced by the type of additive introduced, as well as its percentage in the mixture. With the increased amount of micro- and nanographite additions to the AISI 316L austenitic stainless steel powder, as well as with the increased BET specific surface area of the graphite particles, the total porosity decreases. At the same time, the proportion of the open porosity decreases according to this tendency. The lowest total porosity values were obtained for sinters made from mixtures of the AISI 316L austenitic stainless steel powder with the addition of 0.2% and 0.3% by weight of graphite nanopowder with a BET specific surface area equal to 400 m^2^/g. They amounted to 8% and 7.5%, respectively—with an open porosity of about 2.2% and 1.6%.

### 3.2. Dilatometry (DIL)

**Figure 9:**
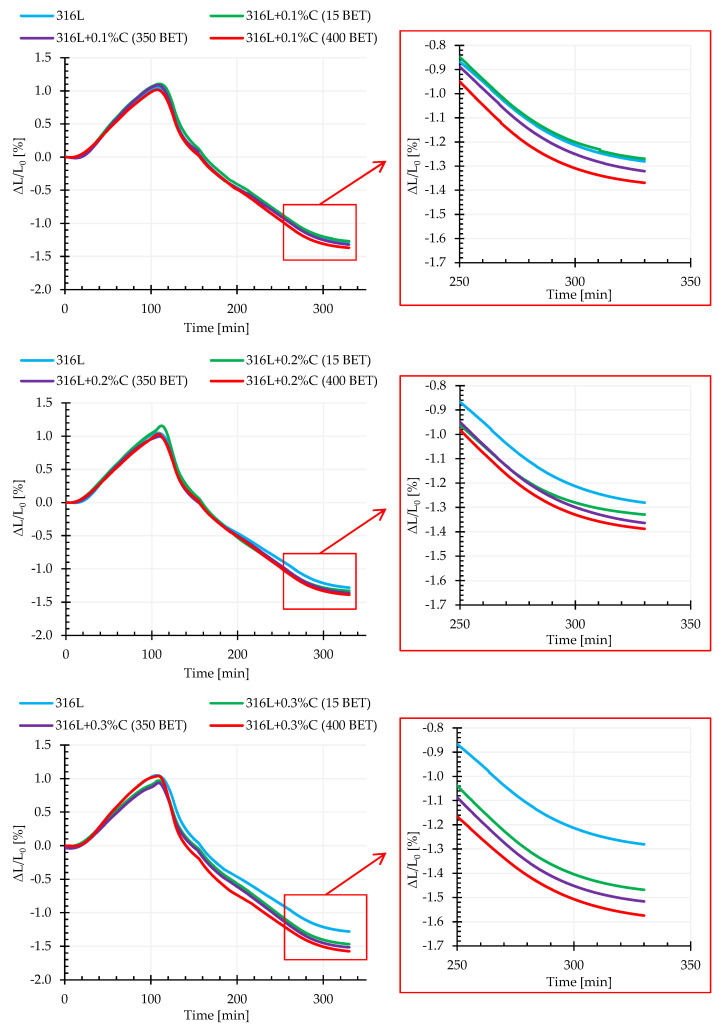
Dilatometric curves of the AISI 316L austenitic stainless steel powder and mixtures of this powder with the addition of 0.1%, 0.2%, and 0.3% by weight micrographite with a BET specific surface area of 15 m^2^/g and nanographites with a BET specific surface area of 350 m^2^/g and 400 m^2^/g (isothermal sintering temperature 1280 °C).

The graphs in Figure 9 show the exemplary dimensional change curves as a function of time for the tested mixtures, for isothermal sintering temperature equal to 1280 °C. Based on the obtained results of dilatometric tests, the values of dimensional changes in individual sintering stages (during heating to isothermal sintering temperature, during isothermal sintering and cooling) were determined, which are summarized in Table 3. 

Analysis of the presented results shows that the addition of micro- and nanographite powders has a significant impact on the sintering process. Up to a certain temperature, in the range of 1050 °C to 1100 °C, the phenomenon of thermal expansion dominates, while above this temperature the samples undergo shrinkage when the sintering processes begin, which indicates the occurrence of mass transport phenomena (evaporation and condensation, surface diffusion, diffusion at grain boundaries, and volume diffusion). As the BET specific surface area increases, as well as the amount of added micro- and nanographite powders to AISI 316L austenitic stainless steel powder, a more intense shrinkage in isothermal sintering can be observed. Most likely, this results from the dissolution of carbon in the matrix material, as well as the intensification of the reduction reaction of the oxide coating, resulting in faster exposure of clean metallic surfaces of powder particles, and as a result acceleration of mass transport phenomena. The addition of graphite also had an impact on the overall dimensional changes of the samples tested—their total shrinkage after sintering increased with the increase of carbon content and the BET specific surface area of graphite particles. Also the temperature of isothermal sintering affected the dimensional changes of the samples—the higher it was, the higher the total shrinkage recorded. It should be remembered that sintering in a acuum entails problems associated with intensive evaporation at high temperatures of elements with a relatively high vapor pressure, which can be included in the tested powder, manganese and chromium—which significantly affects the final sintering effect.

### 3.3. Thermogravimetry (TG)

The thermogravimetric tests combined in one process with calorimetric tests supplemented dilatometric tests and were performed for all tested materials for which isothermal sintering was carried out at three different temperatures, namely 1200 °C, 1240 °C, and 1280 °C. TG thermogravimetric measurements, consisting of measuring the mass changes of the tested powder mixture as a function of time or temperature, allowed the determination of the impact of both the percentage composition of this mixture, as well as the isothermal sintering temperature on the size of recorded thermal transformations and temperatures at which these transformations took place.

**Figure 10:**
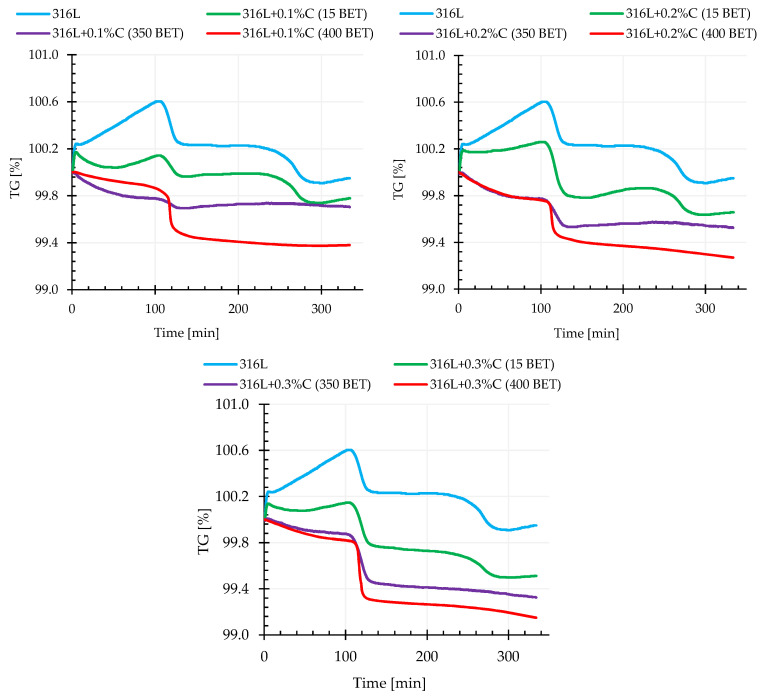
Thermogravimetric curves for the AISI 316L austenitic stainless steel powder and mixtures of this powder with the addition of 0.1%, 0.2%, and 0.3% by weight micrographite with a ET specific surface area of 15 m^2^/g and nanographites with a BET specific surface area of 350 m^2^/g and 400 m^2^/g (isothermal sintering temperature 1280 °C).

Figure 10 shows exemplary thermogravimetric curves recorded during sintering at 1280 °C of the AISI 316L austenitic stainless steel powder and its mixtures with the addition of 0.1%, 0.2%, and 0.3% by weight of graphite powders, characterized by different BET specific surface area of their particles.

Analyzing the obtained thermograms for samples of pure powder of the tested steel and its mixtures with graphite micropowder, it was noticed that in the initial sintering stage, during heating, until the temperature reached about 1000 °C, a mass increase was recorded, which was probably associated with the occurring oxidation of the material. It is worth noting that this phenomenon was not observed in the case of samples made of AISI 316L powder with the addition of graphite nanopowders.

For all mixtures of graphite-doped austenitic stainless steel (irrespective of the BET specific surface area of the powder particles), for a temperature range from about 1000 °C to 1100 °C, there was a sharp decrease in mass. The recorded weight loss of the examined materials was more intense the higher the sintering temperature and the higher the amount of graphite powder addition, and the higher the degree of BET specific surface area development of the graphite particles. 

It can be read from the thermogravimetric curves that for the tested steel powder modified with the addition of 0.1%, 0.2%, and 0.3% by weight graphite micropowder, the weight loss was about 0.2%, 0.4%, and 0.5% respectively. In comparison, for the same weight shares of the addition of graphite nanopowder with a specific surface area of 350 m^2^/g, the recorded weight loss was about 0.5%, 0.7%, and 0.71% respectively. However, the highest weight loss, among the tested materials, was recorded for AISI 316L steel powder doped with graphite nanopowder with the highest BET specific surface area of particles—400 m^2^/g—which for the tested nanographite mass shares, was about 0.6%, 0.7%, and 0.9% respectively. The smallest weight loss was obtained for pure austenitic stainless steel powder—it amounted to 0.05%.

### 3.4. Qquadrupole Mass Spectroscopy (QMS)

It can be assumed that the relationship between the weight loss and the amount and type of graphite powders added to the AISI 316L powder is closely related to the intensification of the carbon oxide reduction reaction occurring during the sintering process. This assumption is confirmed by the results obtained from quadrupole mass spectroscopy (QMS). This analysis allowed us to determine the composition of gaseous products released during sintering of the tested samples. This work focuses on the analysis of mass spectra corresponding to the carbon dioxide CO_2_ (atomic mass 44), the carbon monoxide CO (atomic mass 28), and the carbon C (atomic mass 12) during sintering of the tested graphite doped steel.

**Figure 11:**
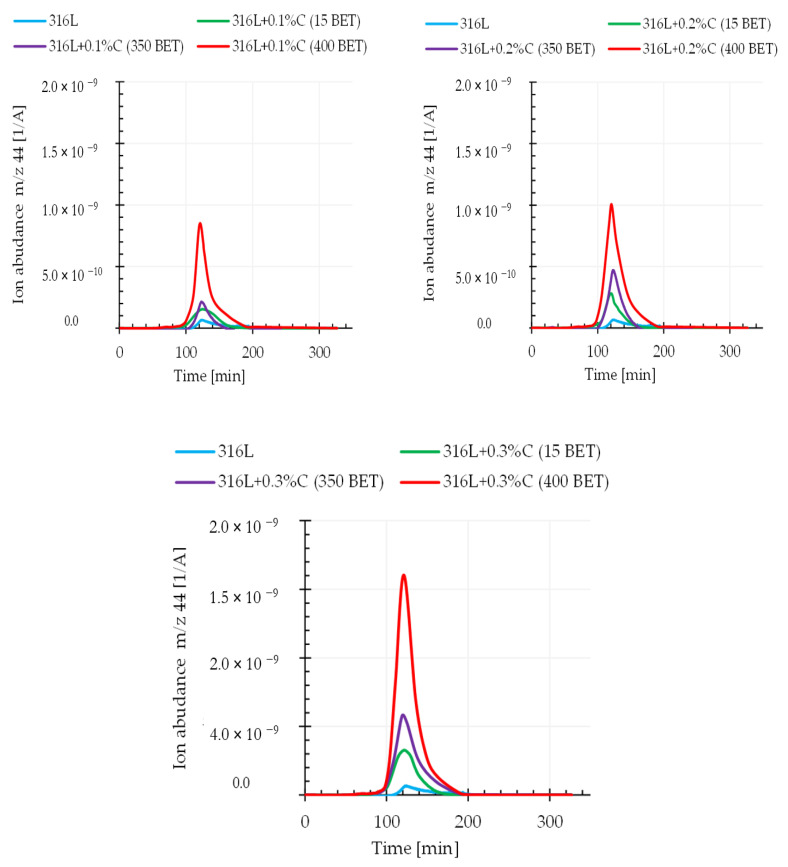
Changes in the emission of gaseous products (m/z 44—CO_2_) registered by QMS for the AISI 316L austenitic stainless steel powder and mixtures of this powder with the addition of 0.1%, 0.2%, and 0.3% by weight micrographite with a BET specific surface area of 15 m^2^/g and nanographites with a BET specific surface area of 350 m^2^/g and 400 m^2^/g (isothermal sintering temperature 1280 °C).

**Figure 12:**
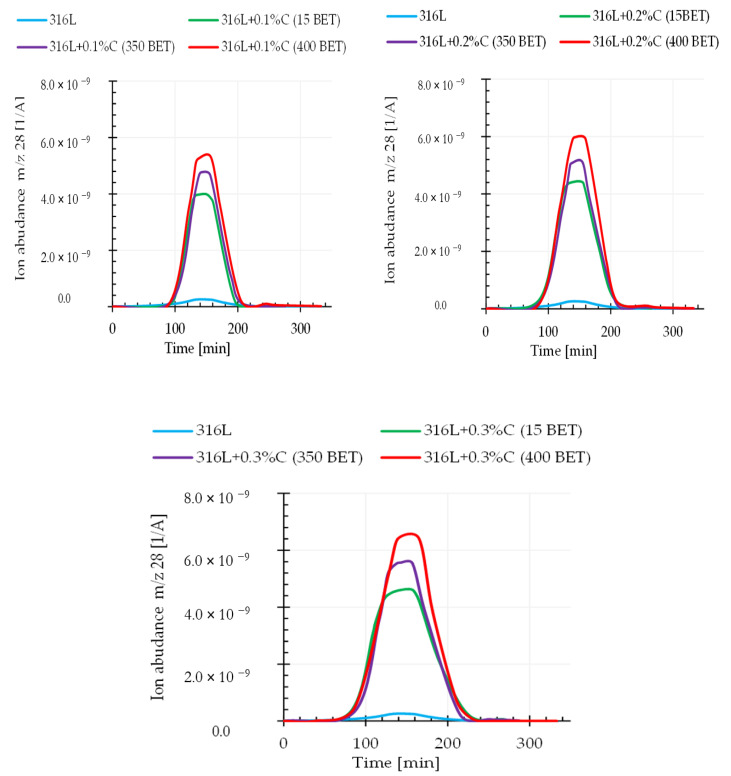
Changes in the emission of gaseous products (m/z 28—CO) registered by QMS for the AISI 316L austenitic stainless steel powder and mixtures of this powder with the addition of 0.1%, 0.2%, and 0.3% by weight micrographite with a BET specific surface area of 15 m^2^/g and nanographites with a BET specific surface area of 350 m^2^/g and 400 m^2^/g (isothermal sintering temperature 1280 °C).

Figure 11 and Figure 12 show exemplary spectra recorded during QMS tests corresponding to atomic mass 44 (CO_2_) and atomic mass 28 (CO) for sintering temperature of 1280 °C.

Analyzing the obtained results, it can be noticed that the intensity of gaseous product evolution from the tested material during the tests largely depended on the sintering temperature as well as on the type and amount of graphite powder additions to the austenitic stainless steel powder. An increase in the emission intensity of the analyzed gaseous products along with increasing graphite powder amount and BET specific surface area as well as with the isothermal temperature of sintering, was observed. This proves that the processes of reducing the oxide layer by carbon were activated, in accordance with the theory presented in the introduction—carbon reduces the oxide layer by forming carbon monoxide (CO), which then allows the reduction of oxides by forming carbon dioxide (CO_2)_. It should be emphasized that the degree of BET specific surface development of graphite powder particles significantly influenced the intensification of these processes. This effect allows faster exposure of clean metallic surfaces of powder particles, resulting in increased sinter density, while reducing their porosity.

### 3.5. Differential Scanning Calorimetry (DSC)

**Figure 13:**
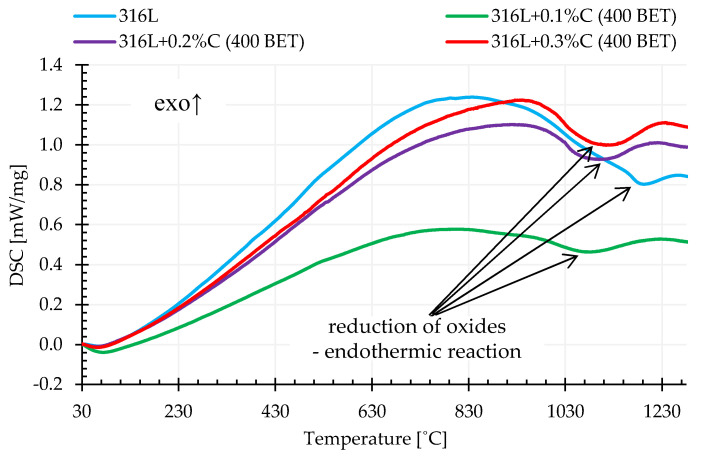
DSC curves for the heating stage to a temperature of 1280 °C for AISI 316L austenitic stainless steel powder and its mixtures with the additions of 0.1%, 0.2%, and 0.3% by weight graphite nanopowder with a BET specific surface area of 400 m^2^/g.

Differential scanning calorimetry (DSC) allowed determination of the influence of the composition of tested mixtures of powders and sintering temperature on the rate of heat supplied to the tested sample in relation to the control sample. Based on the obtained calorimetric curves, it was found that in the temperature range from about 900 °C to 1200 °C, the addition of graphite powder caused an increase in the rate of heat supply to the test sample in relation to the control sample. This is demonstrated by the endothermic peaks visible on the recorded curves. The sizes of these peaks, understood here as the areas under the peaks, were the higher, the higher the isothermal sintering temperature, amount of carbon addition and BET specific surface area of the graphite particles. This is due to the fact that the reaction of metal oxide reduction is endoenergetic, and as demonstrated in the conducted tests, in the case of sintering of AISI 316L powder with the addition of graphite, the reactions of oxide reduction occurred more intensively compared to unmodified steel powder. It is also worth emphasizing that in the case of measurements carried out for loosely filled, unmodified powder of the tested stainless steel, a significant shift of the recorded thermal effect towards higher temperatures and a significant reduction of its intensity in relation to samples with the addition of graphite powders were observed. Figure 13 shows examples of calorimetric curves for the heating stage to a temperature of 1280 °C determined for pure austenitic stainless steel powder and its mixtures with the addition of 0.1%, 0.2%, and 0.3% by weight of graphite nanopowder with a BET specific surface area of its particles equal to 400 m^2^/g.

**Figure 14:**
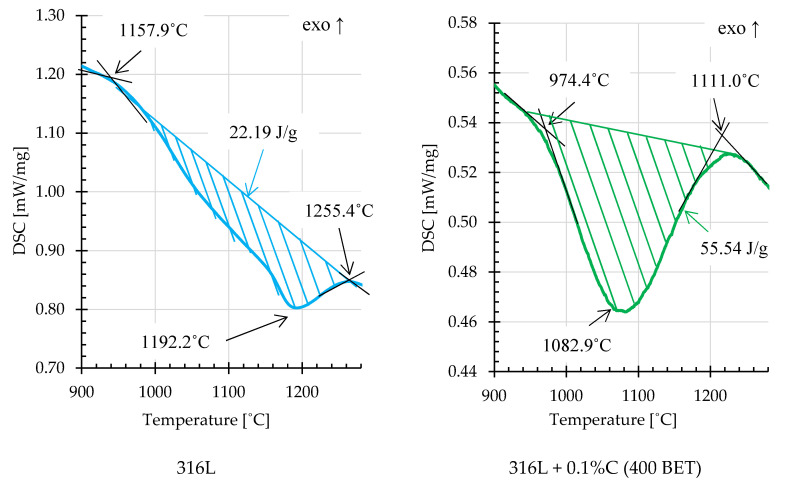
Fragments of DSC curves, illustrating examples of endothermic thermal effects for AISI 316L steel powder and its mixture with the addition of 0.1% wt. graphite nanopowder with a BET specific surface area of 400 m^2^/g (isothermal sintering temperature 1280 °C).

**Figure 15:**
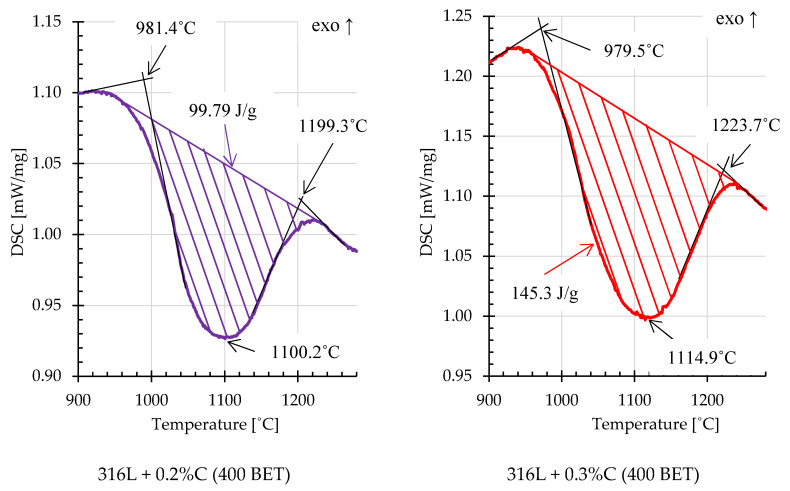
Fragments of DSC curves, illustrating examples of endothermic thermal effects for AISI 316L steel powder with the addition of 0.2% and 0.3% by weight of graphite nanopowder with a BET specific surface area of 400 m^2^/g (isothermal sintering temperature 1280 °C).

On the basis of the obtained DSC curves, the beginning, end and extreme point temperatures were determined for the registered endothermic effects. In order to calculate the energy of these reactions, an interpolated baseline was determined for individual thermal effects, and then the area under the peaks was calculated [34]. Figure 14 and Figure 15 show examples of thermal effects for loosely filled AISI 316L steel powder and mixtures of this powder with the addition of graphite nanopowder with a BET specific surface area of 400 m^2^/g, with characteristic values inscribed. Based on the obtained results, it can be stated that mixtures with the addition of 0.2% and 0.3% by weight of graphite nanopowder with a BET specific surface area of 400 m^2^/g, compared to other samples, were characterized by the highest endothermic reaction energies, respectively about 99,8 J/g and 145.3 J/g. For comparison, the energy of the endothermic reaction recorded on the calorimetric curve obtained for pure AISI 316L steel powder was about 22.19 J/g.

### 3.6. Carbon and Oxygen Content in Sinters

The results of the conducted tests showed that the oxide reduction processes during the sintering process occurred most intensively at a temperature of 1280 °C. The tested sintered materials at this temperature also had the lowest porosity. Therefore tests of carbon and oxygen content in sinters were carried out only for samples sintered at 1280 °C. 

**Figure 16:**
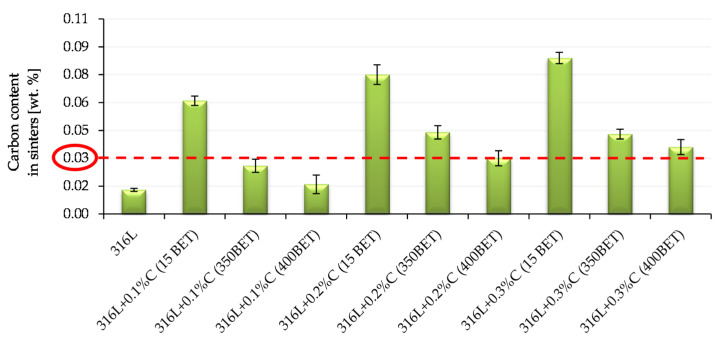
Carbon content in sinters depending on the percentage composition of powder mixtures (isothermal sintering temperature 1280 °C).

**Figure 17:**
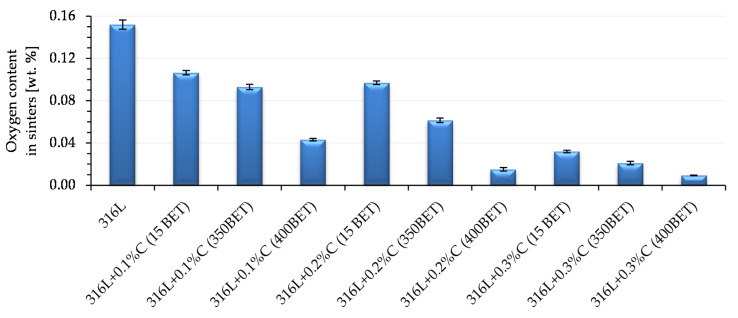
Oxygen content in sinters depending on the percentage composition of powder mixtures (isothermal sintering temperature 1280 °C).

Figure 16 and Figure 17 show the results of measurements of carbon and oxygen content in sinters (standard deviations are marked on the bars presented in Figure 16 and Figure 17). In Figure 16, a red dotted line indicates the content of 0.03% carbon in the sinter—this is the value considered as the limit for the tested steel, after exceeding which there is a risk of reducing the resistance to intergranular corrosion and carbide formation in the steel structure at elevated temperatures.

In the case of sinters made from mixtures of AISI 316L austenitic stainless steel powder with the addition of micro- and nanographite powders, the determined carbon content in the samples after sintering was higher than that obtained for sinters made of pure powder of this steel. On this basis, it can be concluded that the added graphite was not completely removed from the samples during the sintering process. 

The introduction of additives in the form of the micro- and nanographite powders resulted in an increase in carbon content in samples after sintering—the greater the higher proportion of graphite in the mixtures. However, analyzing the obtained results, it can be observed that as the degree of BET specific surface area development of graphite particles increased, the measured carbon content in sinters significantly decreased. The highest carbon content after sintering, among the samples doped with graphite, had sinter made of AISI 316L steel with the addition of 0.3% by weight graphite micropowder with a BET specific surface area of 15 m^2^/g—it amounted to 0.084%. The carbon content in samples after sintering, equal to or not exceeding 0.03%, was characterized by only four of the tested sinters made of the following: pure powder of tested stainless steel and mixtures of this steel with the addition of 0.1% by weight of nanographite powders with a BET specific surfaces area of 350 m^2^/g and 400 m^2^/g and with 0.2% by weight of nanographite with a BET specific surface area of 400 m^2^/g. However, in the case of a sample made of AISI 316L steel with the addition of 0.3% by weight of graphite nanopowder with a BET specific surface area of 400 m^2^/g, the amount of carbon in the sinter slightly exceeded the limit value and amounted to 0.36%. However, it should be emphasized, that for this sinter the largest decrease in carbon content was noted in relation to its initial amount in the mixture from which it was made.

The results of measurements of oxygen content in the sinters tested are shown in Figure 17. The decrease in oxygen content in sinters was the higher the greater the proportion of graphite powder in the mixtures and the BET specific surface area of its particles. The lowest oxygen contents were found in sinters made of mixtures of AISI 316L powder with the addition of 0.2% and 0.3% by weight of nanographite powder with a BET specific surface area of 400 m^2^/g. They amounted to 0.015% and 0.009% respectively.

## 4. Conclusions

In this work, the influence of the modification of AISI 316L austenitic powder stainless steel with the addition of graphite micro- and nanopowders on the sintering kinetics and oxide reduction mechanism was examined. Graphite powders differing in the degree of BET specific surface area of particles were used in the tests. On the basis of the results obtained, the following conclusions can be made:

- Graphite during the sintering process acts as an activator of the reduction reaction of the oxide coating covering particles of the tested steel. Activation of oxide reduction processes during sintering of AISI 316L austenitic stainless steel is significantly influenced by the amount and type of graphite added—the greater the proportion of graphite in the powder mix and the more strongly developed the BET specific surface area of the graphite particles ensure intensification of the oxide reduction process. This is indicated by the determined oxygen content in the samples after sintering. An additional factor affecting the intensification of the oxide reduction processes is the isothermal sintering temperature—the higher it was, the better was the effect of sintering activation obtained for the tested materials. The conducted thermal analysis showed that the samples made of austenitic stainless steel doped with 0.2% and 0.3% by weight graphite nanopowder with a BET specific surface area of 400 m^2^/g, sintered best the oxide reduction reactions, with the participation of carbon, for these samples, reaction occurred the most intensively.
- When designing a sintered material based on AISI 316L steel with the addition of graphite, its quantity should be carefully selected so that it does not exceed 0.03% in the sample after sintering, due to the risk of adverse changes in the steel structure (carbide formation at the grain boundaries reducing the resistance to intergranular corrosion) during other technological processes (e.g., welding) or sinter exposition at elevated temperatures. The obtained results of measurements of carbon content in sinters indicate that the doped graphite is not removed completely from the samples during the sintering process—its admissible amount (0.03%) was kept for four of the tested samples, i.e., for sinters made from pure AISI 316L steel and mixtures of this steel with the addition of: 0.1% by weight of nanographite powders with BET specific surface area of 350 m^2^/g and 400 m^2^/g and 0.2% by weight of nanographite powder with a BET specific surface area of 400 m^2^/g. However, in the case of a sample made of AISI 316L steel doped with graphite nanopowder with a BET specific surface area of 400 m^2^/g—with the amount of 0.3% by weight—the carbon content in the sinter slightly exceeded the limit value and amounted to 0.036%.

## Figures and Tables

**Table 1:** Chemical composition of the AISI 316L austenitic stainless steel powder.

Chemical Element	C	S	Mo	Ni	Mn	Cr	Si	O *	N	Fe
Percentage [% wt.]	0.018	0.01	2.3	12.9	0.1	17.0	0.9	0.14	0.06	Bal.

* Oxygen occurs mainly in the form of oxides formed on the surface of powder particles (defined according to PN-EN ISO 4491-2:2002 [32].

**Table 2:** Composition of prepared mixtures and their designation.

No.	Mixture Composition [% wt.]	Mixture Designation
1	100% AISI 316L	316L
2	99.9% AISI 316L + 0.1% micrographite (BET * specific surface area of 15 m^2^/g)	316L + 0.1%C (15 BET)
3	99.8% AISI 316L + 0.2% micrographite (BET * specific surface area of 15 m^2^/g)	316L + 0.2%C (15 BET)
4	99.7% AISI 316L + 0.3% micrographite (BET * specific surface area of 15 m^2^/g)	316L + 0.3%C (15 BET)
5	99.9% AISI 316L + 0.1% nanographite (BET * specific surface area of 350 m^2^/g)	316L + 0.1%C (350 BET)
6	99.8% AISI 316L + 0.2% nanographite (BET * specific surface area of 350 m^2^/g)	316L + 0.2%C (350 BET)
7	99.7% AISI 316L + 0.3% nanographite (BET * specific surface area of 350 m^2^/g)	316L + 0.3%C (350 BET)
8	99.9% AISI 316L + 0.1% nanographite (BET * specific surface area of 400 m^2^/g)	316L + 0.1%C (400 BET)
9	99.8% AISI 316L + 0.2% nanographite (BET * specific surface area of 400 m^2^/g)	316L + 0.2%C (400 BET)
10	99.7% AISI 316L + 0.3% nanographite (BET * specific surface area of 400 m^2^/g)	316L + 0.3%C (400 BET)

* BET-measurement technique of the specific surface area of materials based on Brunauer–Emmett–Teller theory [31].

**Table 3:** Dimensional changes during the sintering process depending on the percentage composition of mixtures and the isothermal sintering temperature.

Dimensional Changes [%]				
**Sample Designation**	**Heating**	**Isothermal Sintering at 1200 °C**	**Cooling**	**Total Dimensional Changes**
316L	+0.96	−0.56	−1.27	−0.86
316L + 0.1%C (15 BET)	+0.93	−0.48	−1.28	−0.83
316L + 0.1%C (350 BET)	+0.91	−0.55	−1.31	−0.95
316L + 0.1%C (400 BET)	+0.90	−0.65	−1.26	−1.01
316L + 0.2%C (15 BET)	+0.86	−0.53	−1.28	−0.95
316L + 0.2%C (350 BET)	+0.92	−0.67	−1.31	−1.06
316L + 0.2%C (400 BET)	+0.73	−0.56	−1.25	−1.08
316L + 0.3%C (15 BET)	+0.91	−0.58	−1.30	−0.95
316L + 0.3%C (350 BET)	+0.82	−0.63	−1.33	−1.14
316L + 0.3%C (400 BET)	+0.93	−0.72	−1.39	−1.18
**Sample Designation**	**Heating**	**Isothermal Sintering at 1240 °C**	**Cooling**	**Total Dimensional Changes**
316L	+0.90	−0.70	−1.30	−1.10
316L + 0.1%C (15 BET)	+0.92	−0.73	−1.33	−1.14
316L + 0.1%C (350 BET)	+0.82	−0.67	−1.31	−1.16
316L + 0.1%C (400 BET)	+0.78	−0.63	−1.36	−1.21
316L + 0.2%C (15 BET)	+0.72	−0.56	−1.34	−1.18
316L + 0.2%C (350 BET)	+0.79	−0.64	−1.36	−1.21
316L + 0.2%C (400 BET)	+0.74	−0.66	−1.37	−1.29
316L + 0.3%C (15 BET)	+0.64	−0.58	−1.36	−1.30
316L + 0.3%C (350 BET)	+0.67	−0.62	−1.38	−1.33
316L + 0.3%C (400 BET)	+0.65	−0.67	−1.36	−1.38
**Sample Designation**	**Heating**	**Isothermal Sintering at 1280 °C**	**Cooling**	**Total Dimensional Changes**
316L	+0.71	−0.67	−1.33	−1.28
316L + 0.1%C (15 BET)	+0.81	−0.68	−1.40	−1.27
316L + 0.1%C (350 BET)	+0.70	−0.62	−1.37	−1.29
316L + 0.1%C (400 BET)	+0.65	−0.62	−1.40	−1.37
316L + 0.2%C (15 BET)	+0.72	−0.66	−1.39	−1.33
316L + 0.2%C (350 BET)	+0.61	−0.62	−1.35	−1.36
316L + 0.2%C (400 BET)	+0.59	−0.60	−1.38	−1.39
316L + 0.3%C (15 BET)	+0.53	−0.58	−1.43	−1.46
316L + 0.3%C (350 BET)	+0.52	−0.61	−1.43	−1.52
316L + 0.3%C (400 BET)	+0.47	−0.66	−1.39	−1.57
